# Zusanli (ST36) acupoint injection for acute diarrhea in children under 5 years old: A protocol of systematic review and meta-analysis of randomized clinical trials

**DOI:** 10.1097/MD.0000000000016949

**Published:** 2019-08-23

**Authors:** Yuan Tian, Hengchang Hu, Yuan Zhang, Linyue Zhou, Lizhen Wang, Chunguang Xie

**Affiliations:** Hospital of Chengdu University of Traditional Chinese Medicine, Chengdu, Sichuan Province, China.

**Keywords:** acupoint injection, acute diarrhea, children, protocol, systematic review, Zusanli (ST36)

## Abstract

**Background:**

Acute diarrhea is the 2nd highest prevalence disease among children under 5 years of age. It can cause malnutrition and even death in children, especially in developing country. Traditional Chinese medicine therapy has been applied and already in the guidelines for clinical practice of acute infectious diarrhea in children in China, but there is no specific methods or recommendations due to lacking of evidence. Zusanli acupoint injection as a form of acupuncture therapy, which is proved to be effective in randomised controlled trials (RCTs) and very suitable for children, has been used in acute diarrhea in children for a long time; therefore, a systematic review is necessary to provide available evidence for further study.

**Methods:**

Different studies from various databases will be involved in this study. Only RCTs of children under 5 years of age diagnosed with acute diarrhea using any recognized diagnostic criteria will be included. We will search manually the literature in the databases from China Conference Paper Database. Electronic database includes PubMed, Embase, Cochrane Library, Web of Science, China National Knowledge Internet, WanFang, Chongqing VIP, and China Biomedical Literature CDROM Database. Primary outcomes: clinical cure rate (clinical cure is defined as the frequency, timing and character of stool back to normal status, as well as disappearance of diarrhea symptoms), diarrhea duration (from admission to the cessation of diarrhea). Secondary outcomes: stool frequency within 24 hours, rate of adverse effect. Data will be extracted by 2 researchers independently; risk of bias of the meta-analysis will be evaluated based on the Cochrane Handbook for Systematic Reviews of Interventions. All data analysis will be conducted by data statistics software Review Manager V.5.3. and Stata V.12.0.

**Results:**

This study will synthesize and provide evidence based on the data of the currently published zusanli (ST36) acupoint injection for acute diarrhea in children under 5 years old, especially in terms of clinical efficacy and safety.

**Conclusion:**

This systematic review aims to evaluate the benefits and harms of zusanli acupoint injection for acute diarrhea in children under 5 years old reported in RCTs, and provide evidence reference in TCM field for Chinese guidelines on the treatment of acute diarrhea in children.

**Ethics and dissemination:**

This study is a systematic review; the outcomes are based on the published evidence, and hence examination and agreement by the ethics committee are not required in this study. We intend to publish the study results in a journal or conference presentations.

**PROSPERO registration number:**

PROSPERO 2019 CRD42019135275.

## 1 Introduction

### 1.1 Description of the condition

Acute diarrhea is the 2nd highest prevalence disease among children under 5 years of age (YOA).^[1,2]^ It can lead to threatening dehydration and electrolytes disorder, and it is an important cause of malnutrition in children.^[3]^ Acute diarrhea is not only one of the leading causes for hospital attendance of children (more than 50% of hospitalization of acute diarrhea was induced by rotavirus infection), but also one of the leading causes of morbidity and mortality in children younger than 5 YOA. About 577,508 children worldwide die of this disease every year, mainly in developing countries. China is still one of the countries with the highest diarrhea mortality rate. About 9072 children under 5 YOA die of diarrhea every year.^[4]^

### 1.2 Description of the intervention

Routine treatment for acute diarrhea in children, including fluid replacement therapy, dietary therapy, zinc supplementation therapy, drug treatment as probiotics, antibiotics, montmorillonite, Chinese medicine, etc. Acupoint injection involves the injection of medicine into specific acupuncture points to treat diseases or conditions, it is a synergetic effect of acupuncture and medication.^[5,6]^ Acupoint injection also is a very successful treatment method combining traditional Chinese medicine (TCM) and western medicine. Clinical and experimental research shows that acupoint injection has the characteristics of low dosage and quick effect, and it is possible to magnify the pharmacologic effects of drugs by geometric order of magnitude,^[7,8]^ but the mechanism of the effect is still unclear.^[8]^

### 1.3 How the intervention might work

Zusanli (ST36) is an acupoint located below the knee, on the tibialis anterior muscle, along the stomach meridian. We chose this acupoint because it is one of the most commonly used acupoints in Chinese medicine for diseases of spleen-stomach, and hence it is often applied to treat digestive system disease such as vomiting, diarrhea, colitis, dyspepsia, etc.^[9,10]^ Studies have shown that acupuncture stimulation of zusanli can promote gastrointestinal motility by increasing the secretion of Motilin and Gastrin in treating functional gastrointestinal disorders-diarrhea (FGIDs-D),^[11]^ and significantly improve gastrointestinal microcirculation by increasing the secretion of 5-HT in treating FGIDs,^[12]^ and effectively improve the weight growth rate of irritable bowel syndrome, reduce the content of inflammatory factors in colonic mucosa, regulate the expression of nuclear factor kappa B protein in colonic tissue to reduce inflammatory injury.^[13]^ Besides, there are randomized controlled trials (RCTs) published in China have indicated that zusanli acupoint injection as a complementary therapy combine with conventional treatment could improve the cure rate and shorten the course of acute diarrhea in children.^[14–16]^

### 1.4 Why it is important to this review

Zusanli acupoint injection is frequently used clinically in acute diarrhea in children as a complementary therapy, and prove to be more effective than using routine treatment only.^[14–16]^ However, there is no critically appraised evidence as systematic review or meta-analysis of the potential benefits and harms of zusanli acupoint injection for acute diarrhea in children. If zusanli acupoint injection proved to be truly effective and safe in this study, and this method itself is simple to be operated, it could be much easier than ordinary acupuncture in application and promotion worldwide, therefore benefits more people.

### 1.5 Objectives

This review aims to systematically evaluate the benefits and harms of zusanli acupoint injection for acute diarrhea in children under 5 years old reported in RCTs. We look forward to provide evidence reference in TCM field for Chinese guidelines on the treatment of acute diarrhea in children.

## 2 Methods

### 2.1 Study registration

The protocol of the systematic review has been registered in International Prospective Register of Systematic Reviews (PROSPERO), and the registration number is CRD42019135275. This systematic review protocol will be conducted and reported strictly according to Preferred Reporting Items for Systematic Reviews and Meta-Analyses (PRISMA)^[17]^ statement guidelines, and the important protocol amendments will be documented in the full review.

### 2.2 Criteria for considering studies for this review

We will strictly screen studies that meet the following inclusion criteria.

#### 2.2.1 Type of included studies

Only RCTs (except quasi-RCTs and cluster RCTs) will be included. Animal mechanism studies and nonrandomized clinical trials will be excluded. Article that substantially overlaps with another published article in print or electronic media will be excluded. Duplicate publications produced by a single experiment and published as separate papers with different criteria for measuring results, priority will be given to original publications and other publications will be excluded. The language and time of publication will not be restricted.

#### 2.2.2 Participants

Children under 5 YOA diagnosed with acute diarrhea using any recognized diagnostic criteria, dysentery, and drug-relationship diarrhea will be excluded, regardless of sex and types of acute diarrhea.

#### 2.2.3 Interventions and controls

Interventions include zusanli acupoint injection as a complementary therapy combined with conventional treatment. Conventional treatment refers to comprehensive treatment, which is based on the guideline^[18]^ and mainly about fluid replacement therapy to maintain acid-base balance of water and electrolyte; diet therapy; zinc supplementation therapy; and pharmacotherapy like probiotics, montmorillonite, antibiotics, etc. Controls use only conventional treatment. The choice of routine treatment in each RCT does not have to be exactly consistent, but zusanli acupoint injection should be the only difference between interventions and controls. Any type of injected medication will be included.

#### 2.2.4 Type of outcome measures

Primary outcomes: clinical cure rate (clinical cure is defined as the frequency, timing and character of stool back to normal status, as well as disappearance of diarrhea symptoms^[19]^), diarrhea duration (from admission to the cessation of diarrhea).

Secondary outcomes: stool frequency within 24 hours, rate of adverse effect.

The most severe threat posed by diarrhea is dehydration, which is closely related to the time and frequency of diarrhea, and hence we record and contrast diarrhea duration (from admission to the cessation of diarrhea) and stool frequency within 24 hours to measure the curative effect. As for clinical cure rate and rate of adverse effect, they are the most intuitive result evaluation.

### 2.3 Search methods

#### 2.3.1 Search resources

This review will include grey literature sourced from China Conference Paper Database, manual searching. Electronic database includes PubMed, Embase, Cochrane Library, Web of Science, China National Knowledge Internet, WanFang, Chongqing VIP, and China Biomedical Literature CDROM Database will also be searched. We will simply present the search process of the PubMed (Table 1). The data will be searched in English and Chinese databases from their inception to April 2019.

**Table 1:**
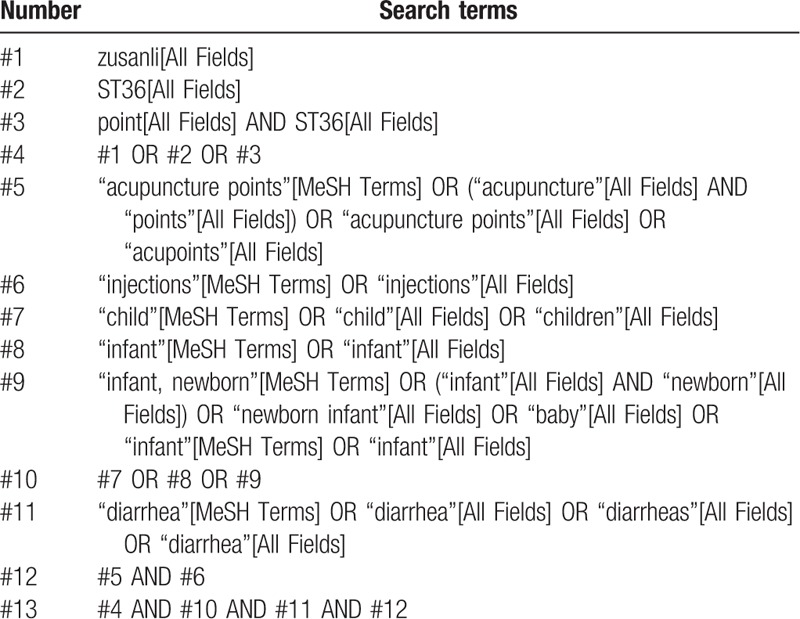
Example of PubMed search strategy.

#### 2.3.2 Search strategies

The following MeSH terms and their combinations will be searched: zusanli OR ST36 OR point ST36; acupuncture points OR acupoints OR points; injections; diarrhea OR diarrheas OR diarrhea; and children OR child OR infant OR baby.

### 2.4 Data collection and analysis

#### 2.4.1 Studies selection

**Figure 1:**
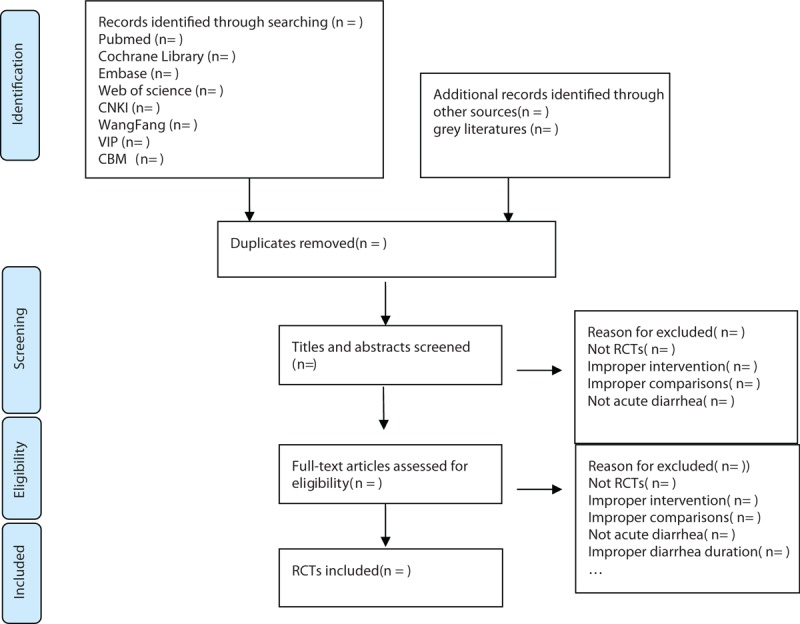
Flow chart of the study selection.

There will be 2 researchers (TY and ZY) carry out the selection of research literature independently using endnote x9 software. We will 1st make the preliminary selection by screening titles and abstracts. Secondly, we will download full text of the relevant studies for further selection according to the inclusion criteria. If there is any different opinion, 2 researchers will discuss and reach an agreement. If a consensus could not be reached, there will be a 3rd researcher (HHC) who makes the final decision. Details of the selection process were shown in the flow chart (Fig. 1).

#### 2.4.2 Data extraction

Two researchers (WLZ and ZLY) will read all the included text in full, and independently extract the following information: basic information (year of publication, the 1st authors name), study method (design, blinding), information of participants (number of children, gender, age, stool frequency within 24 hours, course of disease), information of treatment (interventions and controls, medicine, dose, frequency, duration), outcomes (clinical cure rate, diarrhea duration, stool frequency within 24 hours, rate of adverse effect), and *P*-value. If we could not reach an agreement, a 3rd researcher (HHC) would make the final decision. One researcher (WLZ) would contact the corresponding author by telephone or e-mail for more information when the reported data were insufficient or ambiguous.

#### 2.4.3 Assessment of risk of bias

All the included studies will be evaluated based on the guidelines of Cochrane Handbook for Systematic Reviews of Interventions.^[20]^ The quality of each trial will categorized into “low,” “unclear,” or “high” risk of bias according to the following items: adequacy of generation of the allocation sequence, allocation concealment, blinding of participants and personal, blinding of outcome assessors, incomplete outcome data, selected reporting the results, and other sources of bias (such as comparable baseline characteristic, inclusion and exclusion criteria).

#### 2.4.4 Assessment of reporting biases

Reporting biases and small-study effects will be detected by funnel plot and Egger test if there are 10 more studies included in this meta-analysis. For Egger test, *P*-value of <.10 was considered to indicate the existence of reporting biases and small study effects.

#### 2.4.5 Data analysis

We used RevMan 5.3 software provided by the Cochrane collaboration to analyze the data. Binary outcomes will be summarized using risk ratio with 95% confidence interval (CI) for relative effect. Continuous outcomes will be summarized by using weighted mean difference with 95% CI. We will use random-effect model for meta-analysis in this review according to research recommendations^[21]^.

Statistical heterogeneity will be assessed by Chi-squared and *I*^2^ statistical tests. Where *P*-value ≥.1 and *I*^2^ ≤50%, there is no obvious statistical heterogeneity among the studies. On the contrary, where *P*-value <.1 or *I*^2^ >50% indicates a considerable heterogeneity. Meta-analysis will be performed when the statistical heterogeneity is acceptable (*P*-value ≥.1 and *I*^2^ ≤50%); otherwise, subgroup analysis will be applied to explore the influence of potential factors on the outcome measures. We will conduct subgroup analyses by different medication of injection (654-2, vitamin B, Chinese patent medicine, etc). We will conduct sensitivity analyses by omitting studies one by one to probe the impact of an individual study. If a meta-analysis cannot be performed, we will conduct descriptive analysis instead.

#### 2.4.6 Patient and public involvement

This is a meta-analysis study based on previously published data, and hence patient and public involvement will not be included in this study.

#### 2.4.7 Ethics and dissemination

Ethical approval will not be required as this is a protocol for systematic review and meta-analysis. The findings of this study will be disseminated to a peer-reviewed journal and presented at a relevant conference.

#### 2.4.8 Evidence assessed

The quality of evidence for this study will be assessed by Grades of Recommendations Assessment, Development and Evaluation (GRADE) standard established by the World Health Organization and international organizations.^[22]^ To achieve transparency and simplification, the quality of evidence is divided into 4 levels in GRADE system: high, medium, low, and very low. We will employ GRADE profiler 3.2 for analysis.

## 3 Discussion

Nowadays, acupuncture and moxibustion are considered to be effective in treating gastrointestinal dysfunction. In recent years, more and more studies have found that acupuncture and moxibustion play an important role in regulating gastrointestinal motility,^[23]^ such as diabetic gastroparesis,^[24]^ bowel preexcitation syndrome,^[25]^ etc. especially IBS (diarrhea-predominant irritable bowel syndrome referred to as diarrhea in TCM^[26]^). Recent studies have shown that acupuncture and moxibustion have a definite effect on IBS,^[27]^ acupoints and methods are various,^[28–29]^ and the curative effect is better than that of conventional drugs.^[30]^ Acupuncture and moxibustion treatment of diarrhea-predominant irritable bowel syndrome has obvious advantages in enhancing clinical efficacy, shortening course of treatment, improving main clinical symptoms and serum biochemical indicators more quickly and ideally, and assisting in regulating patients’ mood.^[26]^

Zusanli acupoint has always been the preferred acupoint for the acupuncture therapy of gastrointestinal diseases; therefore, widely used in modern clinical practice in treating gastritis, gastralgia, functional dyspepsia, vomiting, hiccup, abdominal pain, diarrhea, abdominal distention, and other gastrointestinal diseases, with remarkable effective.^[31]^ Studies have proved that acupuncture stimulation of zusanli can enhance gastric motility,^[32,33]^ protect gastric mucosa,^[34]^ and improve intestinal function.^[11–13]^ Acupoint injection therapy can not only exert the function of acupoints, but also exert the pharmacologic function of western medicine; it is a double stimulation with acupuncture and medicine liquid pairs in acupoints.^[4,5,35,36]^ It is difficult for children to adopt acupuncture therapy, and it is also inconvenient for therapist to operate; however, because of the application of acupoint injection, we can achieve the same purpose as acupuncture, which is easy for parents and kids to accept. Therefore, acupoint injection is especially suitable for children.^[37]^

Zusanli acupoint injection combines the triple function of acupuncture, zusanli acupoint, and medicine^[36]^; it was applied in acute diarrhea in children was 1st reported in 1979,^[38]^ and has been used until now. But there is no any systematic review or meta-analysis of the potential benefits and harms on zusanli acupoint injection for acute diarrhea in children, and although TCM treatment is listed in the guidelines for the treatment of acute diarrhea in children, there is also no specific method or recommendation due to lack of evidence.^[18]^ Hence other than providing evidence for acupoint injection of acute diarrhea in children, this study is anticipated to provide evidence reference in TCM field for Chinese guidelines on the treatment of acute diarrhea in children.

In summary, this systematic review and meta-analysis will help to determine potential benefits and harms on zusanli acupoint injection for acute diarrhea in children. Furthermore, the findings of this study may not only provide reference basis for the guideline, but also might promote acupuncture treatment and application of acupuncture points which would benefit more patients in the future.

## Author contributions

**Conceptualization:** Yuan Tian.

**Data curation:** Yuan Zhang, Linyue Zhou, Lizhen Wang.

**Formal analysis:** Yuan Tian, Hengchang Hu.

**Methodology:** Yuan Tian, Hengchang Hu.

**Project administration:** Chunguang Xie.

**Resources:** Yuan Tian, Chunguang Xie.

**Software:** Yuan Tian, Chunguang Xie.

**Writing – original draft:** Yuan Tian.

**Writing – review & editing:** Chunguang Xie.
